# Coil and *n*-butyl-2-cyanoacrylate migration into the stomach after TIPS for gastroesophageal variceal bleeding: a case report and literature review

**DOI:** 10.1186/s13019-022-02062-8

**Published:** 2022-12-10

**Authors:** Yue-Lin Zhang, Chun-Hui Nie, Tan-Yang Zhou, Guan-Hui Zhou, Tong-Yin Zhu, Sheng-Qun Chen, Hong-Liang Wang, Bao-Quan Wang, Zi-Niu Yu, Li Jing, Qi Xia, Hong-Tan Chen, Jun-Hui Sun

**Affiliations:** 1 Hepatobiliary and Pancreatic Interventional Treatment Center, Division of Hepatobiliary and Pancreatic Surgery, The First Affiliated Hospital, Zhejiang University School of Medicine, 79 Qingchun Road, Hangzhou, 310003 Zhejiang Province China; 2 Zhejiang Clinical Research Center of Hepatobiliary and Pancreatic Diseases, Hangzhou, 310003 Zhejiang Province China; 3 Zhejiang Provincial Research Center for Diagnosis and Treatment of Heapatobiliary Diseases, Hangzhou, 310003 Zhejiang Province China; 4 Department of Infectious Disease, State Key Laboratory for Diagnosis and Treatment of Infectious Diseases, National Clinical Research Center for Infectious Diseases, Collaborative Innovation Center for Diagnosis and Treatment of Infectious Diseases, The First Affiliated Hospital, Zhejiang University School of Medicine, Hangzhou, 310003 Zhejiang Province China; 5 Department of Gastroenterology, The First Affiliated Hospital, Zhejiang University School of Medicine, Hangzhou, 310003 Zhejiang Province China

**Keywords:** Transjugular intrahepatic portosystemic shunt (TIPS), Variceal bleeding, Coil, *n*-butyl-2-cyanoacrylate (NBCA), Case report

## Abstract

**Background:**

Transjugular intrahepatic portosystemic shunt (TIPS) is a well-established therapeutic option for the management of variceal hemorrhage in patients with cirrhosis. The simultaneous migration of the coil and *n*-butyl-2-cyanoacrylate (NBCA) is an extremely rare but significant complication after TIPS. Because of its rare presentation, there are currently no definitive recommendations for the management of this condition.

**Case presentation:**

A 46-year-old man with hepatitis B cirrhosis underwent TIPS placement for uncontrolled gastroesophageal varix (GEV) bleeding secondary to portal hypertension in August 2018. During the procedure, large GEVs were embolized using a coil and NBCA. After a year, coil and NBCA migration into the stomach was observed. Attempts to remove the coil using biopsy forceps during esophagogastroduodenoscopy failed. The patient refused further intervention on the coil to prevent further complications and received conservative therapy instead. Close surveillance with endoscopy is recommended for detecting coils and varices.

**Conclusions:**

The present case reports an extremely rare but significant complication after TIPS, which highlights the management and follow-up recommendation for such rare complications. Our experience may provide guidance for the management of future similar cases and stimulate discussion about treatment methods of similar patients.

## Background

Hemorrhage from gastroesophageal varices (GEVs) is a life-threatening sequela of portal hypertension and a major cause of mortality in patients with liver cirrhosis [1, 2]. The transjugular intrahepatic portosystemic shunt (TIPS), as a second line treatment in GEVs bleeding after failure of endoscopic treatment or in case of preemptive TIPS can be performed together with endoscopic band ligation, is a well-established therapeutic option for the management of variceal hemorrhage in patients with cirrhosis [3, 4]. Adjunct embolization of GEVs using coil and *n*-butyl-2-cyanoacrylate (NBCA) could be useful at the time of TIPS insertion to reduce bleeding of GEVs [5]. The simultaneous coil and NBCA migration into the stomach after TIPS is extremely rare. Because of the lack of clinical reports on this condition exist, there are currently no recommended definitive treatments for such cases. Herein, we report one case admitted to our department in August 2018 with a subsequent review of the literature.

## Case presentation

A 46-year-old male patient was admitted to our hospital with repeated melena for the past month. The clinical abdominal examination on admission revealed a soft abdomen. The Murphy sign and shifting dullness were negative. Percussive examination in spleen area showed that the lower margin of the dullness boundary was located 3 cm below the costal margin. The patient had had hepatitis B cirrhosis for more than 10 years and took oral entecavir (0.5 mg, Squibb, Shanghai, China) daily as antiviral therapy. One month ago, the patient was treated by endoscopy at the local hospital due to melena caused by GEVs bleeding, but the effect was limited because of severe and extensive GEVs, and the patient had repeated melena again. After admission, routine blood examination revealed leukopenia 1.7 × 10^9^/L, mild low hemoglobin 112 g/L and thrombocytopenia 30 × 10^9^/L. The blood biochemistry tests showed a slightly elevated total bilirubin of 25.1 μmol/L (normal range < 21 μmol/L). The prothrombin time was slightly increased at 14.9 s. HBsAg and HBcAb results were positive. Hepatitis B virus DNA was increased at 1.81 × 10^2^ IU/mL. Contrast-enhanced computed tomography (CT) of the abdomen indicated hepatic cirrhosis, portal hypertension (the main portal vein was dilated with a diameter of 18 mm), splenomegaly and gastroesophageal varices. The final diagnosis of the presented case were GEVs bleeding secondary to portal hypertension, hepatic cirrhosis and splenomegaly.

**Fig. 1:**
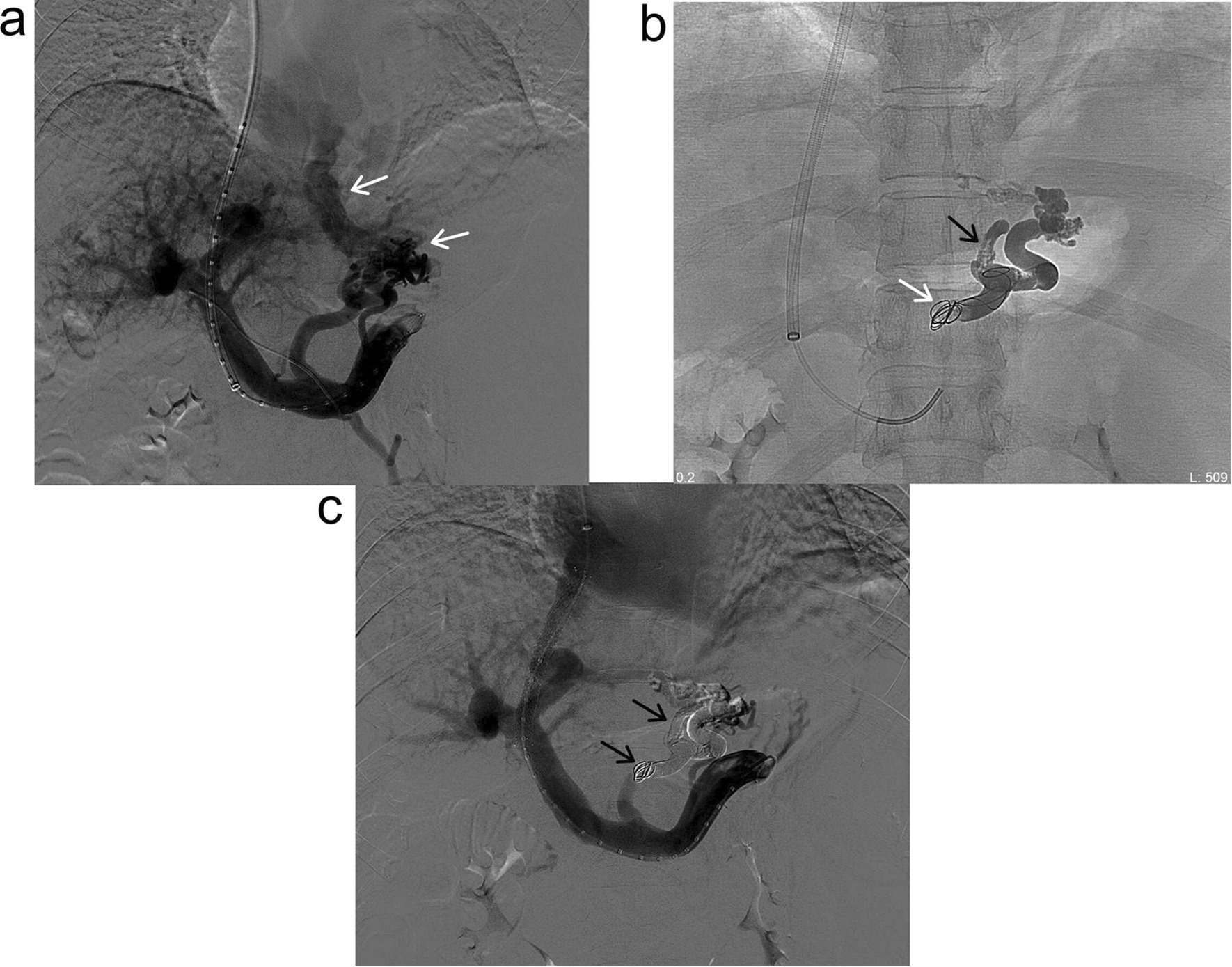
Large GEVs were embolized during the TIPS procedure. **a** Portal venogram demonstrating the GEVs supplied via the splenic vein (white arrow). **b** The GEVs were managed with a proximal detachable coil (white arrow) and distal NBCA (black arrow) embolization. **c** Portal venogram performed after TIPS creation showed complete occlusion of the GEVs (black arrow). GEVs, gastroesophageal varices; TIPS, transjugular intrahepatic portosystemic shunt; NBCA, *n*-butyl-2-cyanoacrylate

The patient underwent TIPS placement for uncontrolled bleeding following a multidisciplinary expert consultation in August 2018. The portal venogram displayed a large drainage into the GEVs from the splenic vein (Fig. 1a). After identification of the GEVs, a soft detachable coil (Boston Scientific, Natick, USA) and NBCA (Histoacryl, Braun, Germany) were deployed for safe and complete embolization during TIPS (Fig. 1b). Subsequently, an intrahepatic shunt tract was created with an 8-mm-diameter covered stent (Gore, Newark, USA) and an 8-mm-diameter bare stent (Boston Scientific, Natick, USA) between the right hepatic and portal veins. The TIPS was performed successfully. The pre-TIPS portosystemic pressure gradient (PSG) was 20 mmHg, and the final PSG was 8 mmHg. The postinterventional venogram showed complete occlusion of the GEVs (Fig. 1c). The patient tolerated the procedure well, and no immediate complications occurred. Hemostasis was also achieved.

**Fig. 2:**
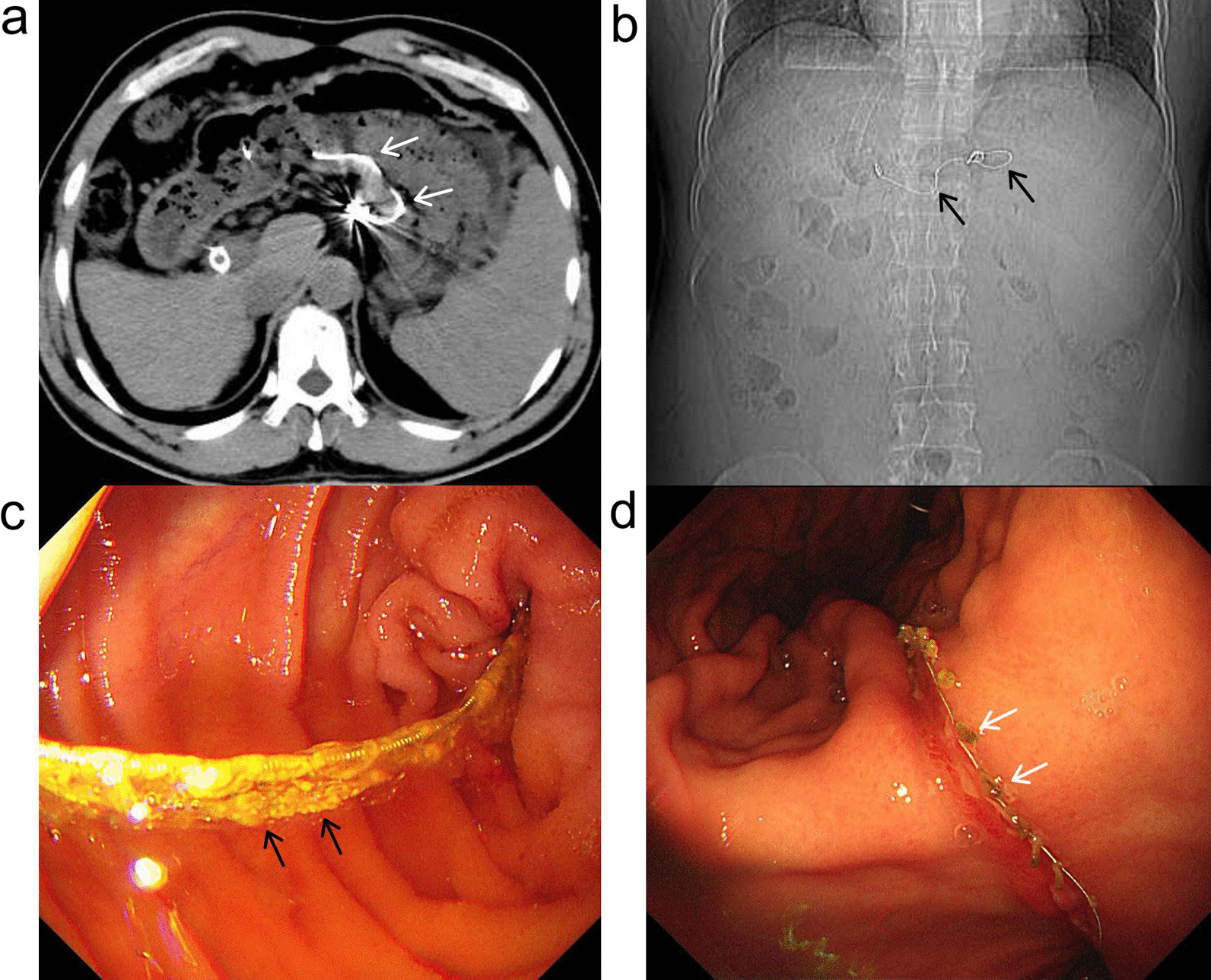
CT and EGD follow-up after TIPS. **a** Abdominal axial CT demonstrated the coil from the GEVs protruding into the stomach (white arrow). **b** Digital scout image revealing migration of the coil (black arrow). The coil was straightened, and NBCA was not visualized. **c** EGD view of the coil and *n*-butyl-2-cyanoacrylate migrating into the gastric wall. Adhesion of the remnants of NBCA at the surface of the coil (black arrow). **d** The coil extended through the gastric wall, resulting in multiple incarcerated ulcers (white arrow). CT, computed tomography; EGD, esophagogastroduodenoscopy; GEVs, gastroesophageal varices; NBCA, *n*-butyl-2-cyanoacrylate

One year after TIPS creation, a follow-up computed tomography (CT) scan showed that a portion of the variceal coil had migrated into the gastric lumen through the site of the previously embolized gastric varix (Fig. 2a), and the NBCA was not seen in the position of the former GEVs on the scout images (Fig. 2b). Subsequent esophagogastroduodenoscopy (EGD) revealed coil erosion through the stomach wall with no active bleeding (Fig. 2c). However, the patient remained asymptomatic. In March 2021, the patient was again admitted to our hospital because of melena. An EGD was performed, which showed the endovascular coil extending through the gastric wall with multiple incarcerated ulcers (Fig. 2d). During the EGD, attempts to remove the coil using biopsy forceps failed. The patient refused intervention on the coil to prevent further complications since his melena symptoms improved after conservative treatment. Our plan is to repeat EGD after 3–6 months for surveillance of the coil and varices.

This study was approved by the ethics committee of The First Affiliated Hospital, College of Medicine, Zhejiang University. Written informed consent was obtained from the patient for publication of this case report and any accompanying images.

## Discussion and conclusions

NBCA is a liquid adhesive agent that can rapidly induce the process of polymerization and solidify when it comes in contact with solutions (such as blood) that contain anions, thereby promoting vessel obliteration, inflammation, and fibrosis [6]. Because of the high risk of pulmonary embolism, coils were used to slow down the blood flow during the embolization of varicose veins [7]. The migration of both the coil and NBCA is an extremely rare complication after TIPS, with only one case having been reported (Table 1). Kupkova et al. [8] incidentally found a metallic coil and NBCA that penetrated into the stomach three weeks after TIPS. Eleven months after TIPS, the coil spontaneously dropped from the gastric varices into the stomach without bleeding symptoms. In our case, the late-onset migration of the coil and NBCA occurred a year after TIPS, and the primary reason for migration in our patient may have differed from that of Kupkova et al. In our patient, the migration was most likely related to the recurrence of portal hypertension caused by the progression of portal thrombosis [9, 10]. The variceal lumen might have widened due to recurrent portal hypertension, leading to migration of the coil and NBCA. Secondly, it is possible that the migration of coils and NBCA can be favorite by the size of coils and amount of NBCA. In addition, the immune response is a potential promigratory factor, as it functions to eliminate and isolate foreign material. Adhesion of macrophages at the surface of the foreign body and formation of a fibrous capsule with the release of degradation mediators are important components of the immunologic response [11]. Local inflammation and fistula formation may have also played a role [12].

**Table 1:** Summary of the cases of coil and NBCA migration following TIPS

Author/Date [Ref]	Age/Sex (y)	Previous liver disease	Vascular abnormality	Site of migration	Migration time from TIPS	Management	Outcome
Kupkova et al. [8]	68/F	Liver cirrhosis due to hereditary haemochromatosis	Gastric varices	Stomach fundus	3 weeks	None	Well
Present case	46/M	Hepatitis B cirrhosis	Gastroesophageal varices	Stomach fundus	1 year	None	Well

*TIPS* Transjugular intrahepatic portosystemic shunt; *NBCA* *n*-butyl-2-cyanoacrylate

Separate coil migration and penetration following TIPS placement are also rarely documented, with only seven cases having been reported in the English literature (Table 2) [9, 10, 12–16]. Because of the rarity of such complications in the literature, it is difficult to make recommendations for the management of this condition. However, considering the different clinical evolutions of such cases, each patient requires an individual assessment. Three case reports have described coil migration without active bleeding following TIPS creation. No further intervention was performed for the migrated coils [12–14]. Merchant et al. [9] reported a case of coil erosion through a gastric varix into the gastric lumen without active hemorrhage after liver transplantation. The migrated coil was left in situ. Unfortunately, the patient died of progressive dyspnea and subsequently developed polymicrobial sepsis. Similarly, another case reported coil penetration into the stomach after liver transplantation. Urgent laparotomy and partial gastrectomy were performed because of massive hematemesis. Unfortunately, the patient died two days after the surgery [15]. Lucas et al. [10] described a case of gastrointestinal hemorrhage due to coil extrusion after TIPS creation in a patient treated with sorafenib. The portal stent was repermeabilized, and the gastric varices were embolized using a plug. Another report described embolization coil erosion through the duodenal varix following TIPS creation. The penetrating coil was removed using endoscopic forceps [16]. In our case, the patient refused further intervention to retrieve the coil and instead received conservative therapy. Appropriate treatment should be given once signs of bleeding are noted to ensure a good prognosis for the patient. Therefore, close surveillance with endoscopy is recommended for detecting the coil and varices.

**Table 2:** Summary of the cases of separate coil migration following TIPS

Author/Date [Ref]	Age/Sex (y)	Previous liver disease	Vascular abnormality	Number of coils insertion	Site of coil migration	Time from coil insertion	Management	Outcome
Lucas et al. [10]	49/F	Alcoholic and hepatitis C cirrhosis	Gastric varices	2	Stomach fundus	2 years	Portal stent was repermeabilized and the gastric varice was embolized using a plug	Well
Hussain et al. [12]	62/M	Hepatitis C cirrhosis	Gastric varices	3	Stomach fundus	2 weeks	None	Well
Merchant et al. [9]	55/F	Hepatitis C cirrhosis	Gastric varices	4	Stomach fundus	Approximately 3 years	None	Died
Oza et al. [13]	61/F	Hepatitis C cirrhosis	Duodenal varices	14	Duodenal lumen	3 months	None	Well
Levi et al. [15]	64/F	Hepatitis C cirrhosis	Gastric varices	Unknown	Stomach fundus	Approximately 3 years	Surgical removal (partial gastrectomy)	Died
Soape et al. [16]	65/F	Primary biliary cholangitis and decompensated cirrhosis	Duodenal varices	Unknown	Cecum	1 month	Endoscopic removal	Well
Pusateri et al. [14]	58/M	Alcoholic cirrhosis	Gastric varices	Unknown	Stomach fundus	Approximately 2 years	None	Well

*TIPS* Transjugular intrahepatic portosystemic shunt

There are currently no recommended definitive treatments for such cases. However, from our experience, we may be able to provide information for future cases, as this complication should be based on individual patient assessments, such as in our case. Although the outcome of this complication is unknown to date, it may potentially contribute to rebleeding or gastric perforation [14]. Furthermore, there is a likelihood of spontaneous dropout of the coil, similar to that of Kupkova et al. Because the migration of the coil and NBCA in our patient was likely related to recurrent portal hypertension, long-term follow-up is mandatory not only to detect such late complications as soon as possible but also to evaluate the progression of cirrhosis, stent patency, and portal thrombosis. Nonetheless, when such complications occur, it is essential to consider the case thoroughly, react appropriately, and learn from the experience [17].

The current case presents an extremely rare but significant complication after TIPS. Our report highlights the management and follow-up recommendation for such rare cases. Since this is only the second case of simultaneous migration of a coil and NBCA, our experience may provide guidance for the management of future similar cases and stimulate discussion about treatment methods of similar patients.

## Data Availability

The datasets supporting the conclusions of this article are included in the article.

## Abbreviations

CT: Computed tomography
EGD: Esophagogastroduodenoscopy
GEVs: Gastroesophageal varices
NBCA: *N*-Butyl-2-cyanoacrylate
PSG: Portosystemic pressure gradient
TIPS: Transjugular intrahepatic portosystemic shunt
